# Myocardial extracellular volume by T1 mapping: a new marker of arrhythmia in mitral valve prolapse

**DOI:** 10.1186/s12968-021-00797-2

**Published:** 2021-09-13

**Authors:** Anna Giulia Pavon, Dimitri Arangalage, Patrizio Pascale, Sarah Hugelshofer, Tobias Rutz, Alessandra Pia Porretta, Mathieu Le Bloa, Olivier Muller, Etienne Pruvot, Juerg Schwitter, Pierre Monney

**Affiliations:** 1 Department of Cardiology, Lausanne University Hospital (CHUV), Rue du Bugnon 46, 1011 Lausanne, Switzerland; 2 Center for Cardiac Magnetic Resonance of the CHUV (CRMC), Lausanne University Hospital, Lausanne, Switzerland; 3 University of Lausanne (UniL), Lausanne, Switzerland; 4 Division of Cardiology, Fondazione Cardiocentro Ticino, Via Tesserete 48, CH-6900 Lugano, Switzerland

**Keywords:** Mitral valve prolapse, Cardiovascular magnetic resonance, Mitral annular disjunction, Interstitial fibrosis

## Abstract

**Objectives:**

We aimed to evaluate the relationship between mitral annular disjunction (MAD) severity and myocardial interstitial fibrosis at the left ventricular (LV) base in patients with mitral valve prolapse (MVP), and to assess the association between severity of interstitial fibrosis and the occurrence of ventricular arrhythmic events.

**Background:**

In MVP, MAD has been associated with myocardial replacement fibrosis and arrhythmia, but the importance of interstitial fibrosis remains unknown.

**Methods:**

In this retrospective study, 30 patients with MVP and MAD (MVP–MAD) underwent cardiovascular magnetic resonance (CMR) with assessment of MAD length, late gadolinium enhancement (LGE), and basal segments myocardial extracellular volume (ECVsyn). The control group included 14 patients with mitral regurgitation (MR) but no MAD (MR-NoMAD) and 10 patients with normal CMR (NoMR-NoMAD). Fifteen MVP–MAD patients underwent 24 h-Holter monitoring.

**Results:**

LGE was observed in 47% of MVP–MAD patients and was absent in all controls. ECVsyn was higher in MVP–MAD (30 ± 3% vs 24 ± 3% MR-NoMAD, p < 0.001 and vs 24 ± 2% NoMR-NoMAD, p < 0.001), even in MVP–MAD patients without LGE (29 ± 3% vs 24 ± 3%, p < 0.001 and vs 24 ± 2%, p < 0.001, respectively). MAD length correlated with ECVsyn (rho = 0.61, p < 0.001), but not with LGE extent. Four patients had history of out-of-hospital cardiac arrest; LGE and ECVsyn were equally performant to identify those high-risk patients, area under the receiver operating characteristic (ROC) curve 0.81 vs 0.83, p = 0.84). Among patients with Holter, 87% had complex ventricular arrhythmia. ECVsyn was above the cut-off value in all while only 53% had LGE.

**Conclusion:**

Increase in ECVsyn, a marker of interstitial fibrosis, occurs in MVP–MAD even in the absence of LGE, and was correlated with MAD length and increased risk of out-of-hospital cardiac arrest. ECV should be includedin the CMR examination of MVP patients in an effort to better assess fibrous remodelling as it may provide additional value beyond the assessment of LGE in the arrhythmic risk stratification.

**Supplementary Information:**

The online version contains supplementary material available at 10.1186/s12968-021-00797-2.

## Introduction

With a prevalence of about 2% in the western population, mitral valve prolapse (MVP) is a relatively common condition, associated with a good overall prognosis [1–3]. However, recent findings suggest an association between MVP and ventricular arrhythmias (VA) as well as an increased incidence of sudden cardiac death (SCD); hence, the terms “arrhythmic” or “malignant” MVP have been coined [4–6]. The nature of this relationship remains only partially deciphered and identifying prognostic factors and mechanisms associated with these events is currently emerging as a challenging task [2, 7, 8]. Several prognostic factors have been reported in the literature, including negative T waves, bileaflet prolapse with leaflet redundancy [1, 8], polymorphic premature ventricular contraction (PVC) [2], flail mitral valve leaflet, significant mitral regurgitation (MR) [9], and most interestingly left ventricular (LV) fibrosis and mitral annular disjunction (MAD) which seem to be key predictors of VA [6, 8, 10–12]. From a pathophysiological perspective, the excessive mobility of mitral valve leaflets may induce mechanical stretch of the inferobasal wall and of the papillary muscles leading to LV fibrosis. Fibrosis can be detected by late gadolinium enhancement (LGE) using cardiovascular magnetic resonance (CMR) and typically involves the papillary muscles and the LV basal inferior segment in the vicinity of the prolapsing posterior mitral leaflet [5, 10].

Myocardial extracellular volume (ECV) quantification by T1 mapping has emerged as an accurate tool to detect ECV expansion, a quantitative marker of diffuse myocardial fibrosis in ischemic and non-ischemic cardiomyopathies [13, 14]. Most importantly, ECV has been found to be an independent predictor of adverse outcome in patients with significant aortic stenosis [15, 16]. In patients with MR, abnormal native T1 mapping indices and shorter post-contrast T1 times have been reported, but the clinical significance of these observations and their association with an increased risk of SCD or arrhythmia remain unclear [17–20].

Herein, we aimed to evaluate the relationship between MAD severity and myocardial interstitial fibrosis at the LV base quantified by T1 mapping in patients with bileaflet MVP. In addition, we sought to assess the association between a history of out-of-hospital cardiac arrest (OHCA) and the extent of interstitial myocardial fibrosis, as well as the relation between myocardial fibrosis and the occurrence of VA on holter monitoring in MVP patients.

## Methods

### Study population

From the CMR registry of the Lausanne University Hospital, we retrospectively included patients with a bileaflet MVP and MAD between January 2011 and October 2019. MVP was defined as a systolic excursion of the mitral leaflets > 2 mm behind the mitral annular plane in the long axis view, i.e. a displacement of > 2 mm into the left atrium (LA) [21]. MAD was defined as the apparent systolic separation of the mitral leaflet insertion from the ventricular myocardium [10]. As a control group, we included patients with various degrees of MR identified by echocardiography, who underwent CMR during the same period. Patients with myocardial infarction, myocarditis, hypertrophic cardiomyopathy, infiltrative heart disease, more than mild associated valvular heart disease and/or a LV ejection fraction (LVEF) < 50% were excluded. Three groups were finally constituted: (1) patients with MVP and MAD (MVP–MAD), (2) patients with MR but without MAD (MR-No MAD), and (3) patients with neither MR nor MAD (No MR-No MAD). Clinical characteristics were collected based on institutional medical records. The CMR registry was approved by the local ethics committee CER-VD (Protocol number 2018-00656) and the patients provided written informed consent for their inclusion.

### Cardiovascular magnetic resonance protocol

Electrocardiogram (ECG)-gated CMR imaging was performed using a 1.5T CMR system (MAGNETOM Aera or Sola, Siemens Healthineers, Erlangen-Germany) with a 32-channel phased-array surface receiver coil. Cine images were acquired using a breath-hold balanced steady-state free precession sequence (bSSFP) in long-axis (2-chamber, 3-chamber and 4-chamber) and short-axis views (8 mm slices without gap, 10–15 slices). Breath-hold ECG-triggered phase contrast velocity sequences for ascending aortic flow evaluation were acquired to estimate the aortic forward flow and calculate the regurgitation fraction. For pre-contrast T1 mapping, we used the ECG-triggered modified Look-Locker inversion recovery (MOLLI) sequence (using the scheme 3(3)3(3)5) on a single short-axis basal LV slice. The basal slice was defined as the slice position of the most basal cine bSSFP slice, where a complete ring of myocardium was visible throughout diastole and systole. Ten minutes after the administration of a 0.2 mmol/kg intravenous bolus of Gadobutrol (Gadovist, Bayer Healthcare, Berlin, Germany), LGE images were acquired using a 2D breath-hold phase-sensitive segmented inversion-recovery gradient echo pulse sequence in the same orientations as cine images. Inversion-time was individually optimized to null normal myocardium. Post-contrast T1-mapping was acquired following LGE imaging (typically 20 min after Gadobutrol bolus injection) using a MOLLI sequence (4(1)3(1)2 scheme). As previously reported in literature [17], pre and post-contrast T1 mapping was optimized to improve the precision of the measurement, in particular pre-contrast T1 mapping was optimized for measuring long T1s of the order of 1000 ms while the post contrast sequence was optimized for short T1s of the order of 200 ms.

### Image analysis

**Fig. 1:**
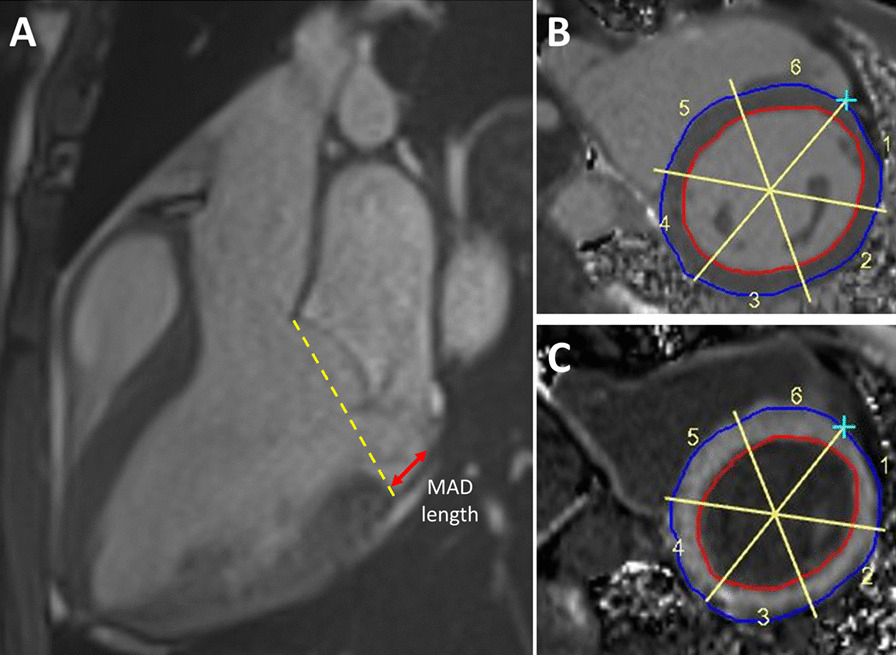
**A** Cardiovascular magnetic resonance (CMR) 3 chambers cine image at end-systole for measurement of mitral annular disjunction (MAD) length. **B**, **C** Semi-automatic measurement of segmental pre-contrast (**B**) and post-contrast (**C**) T1 relaxation time of the basal left ventricular myocardium

All CMR examinations were analyzed blinded to clinical characteristics and outcome using the Syngo.via software (Siemens Healthiners) to calculate LV volumes, LV mass and LVEF by delineating endocardial and epicardial borders in the stack of short-axis cine images. The presence of myocardial LGE was visually evaluated and its extent was semi-quantitatively reported according to the American Heart Association 17 segments model [22]. In the MVP–MAD group, the MR volume was calculated according to the standard indirect method, by subtracting the forward aortic flow volume from the total LV stroke volume (LV stroke volume − forward aortic flow). The MR fraction was quantified using the following equation: (regurgitant volume × 100)/(LV stroke volume). MR volume and regurgitant fraction corrected for the severity of the prolapse were additionally calculated as previously reported [23]. The MAD length was measured on a 3-chamber view from the LA wall-posterior mitral leaflet junction to the top of the basal LV inferolateral wall during systole (Fig. 1, Panel A).

### T1 mapping

Pre- and post-contrast T1 mapping images were first visually reviewed to assess quality, and myocardial T1 relaxation times were then measured using the GTVolume software (GyroTools LLC, Zurich, Switzerland). After manual tracing of the endocardial and epicardial borders, the basal slice of the LV was automatically divided into 6 equal segments and the mean T1 relaxation time was calculated for each segment (Fig. 1, Panel B, C). The synthetic (ECVsyn) was calculated without haematocrit sampling as recently validated by Treibel et al. [24] In particular for ECV calculation the following formula was used [25]:

$${\text{ECV}} = \left( {1 - haematocrit} \right){ } \times { }\frac{{\left( {\frac{1}{{T1{ }myocardial\,post}}} \right) - \left( {\frac{1}{{T1{ }myocardial\;pre}}} \right)}}{{\begin{array}{*{20}c} {\left( {\frac{1}{{T1{ }blood\;post}}{ }} \right) - \left( {\frac{1}{{T1{ }blood\;pre}}} \right)} \\ \end{array} }}$$

According to the literature, synthetic haematocrit was previously validated against conventional haematocrit. For Siemens Aera, a local calibration of the synthetic haematocrit was performed on a population of 20 healthy subjects and more than 800 patients. Following the change in scanner during study period, the local calibration of the synthetic haematocrit was repeated for Siemens Sola on a population of 256 patients. In both cases, a linear regression analysis was used to create a formula allowing synthetic haematocrit estimation from the measured blood relaxivity (R1) (Additional file 1: Figure S1). ECVsyn was then calculated using synthetic haematocrit instead of measured haematocrit in the formula.

ECVsyn expansion was considered to be significant with an ECVsyn > 27%. The reproducibility of T1 relaxation time and ECVsyn measurement was assessed on a random sample of 15 patients, analyzed by two different operators, blinded to clinical data and both experienced in CMR examination analysis.

### Objectives and endpoints definitions

The main objective of the study was to compare the severity of myocardial fibrosis (extent of LGE, pre-contrast T1 relaxation time and ECVsyn) at the basal LV myocardium and the length of MAD in MVP patients with and without a recent history of unexplained resuscitated OHCA. As a secondary objective, we assessed the relation between the severity of myocardial fibrosis and the incidence of PVC and of complex PVC (grade 4 or 5 according to Lown classification) in a subgroup of 15 patients with suspected arrhythmic MVP, who performed a 24 h Holter monitoring.

### Statistical analysis

Continuous variables were expressed as mean ± SD or median [interquartile range] where appropriate, and categorical variables as numbers and percentages (n, %) respectively. Gaussian distribution was determined with the skewness and kurtosis test for normality. Differences in continuous variables between two groups were analysed with the independent-sample Student’s t test in case of normal distribution or the Mann–Whitney test in case of non-normal distribution. Comparisons between three groups were performed by ANOVA with post-hoc Bonferroni correction. Comparison of categorical variables was performed with the χ^2^ test. Correlation analysis was performed using Spearman’s correlation. Receiver operating characteristic (ROC) analysis was used to determine the optimal cut-off value of selected CMR parameters (LGE and ECVsyn) associated with arrhythmic endpoints. Considering the inhomogeneous distribution of pre-contrast T1 relaxation times and ECVsyn, with the highest values typically located in the inferior and inferolateral region (i.e. at the close vicinity of the posterior mitral leaflet attachment), the average values of the basal inferior and inferolateral segments were used for association analyses with arrhythmic endpoints.

Reproducibility analyses for the measurement of myocardial pre-contrast T1 and ECVsyn were performed using Pearson’s correlation and Bland–Altman statistics. All statistical analyses were performed using STATA 16.0 (StataCorp, College Station, Texas, USA). A p < 0.05 was considered statistically significant.

## Results

A total of 38 patients with MVP and MAD were identified. Five patients with complex congenital heart disease and 3 patients who already underwent mitral valve repair were excluded leaving 30 patients in the MVP–MAD group. Thirty-eight patients with various degrees of MR detected by echocardiography and without MAD where selected as controls. Four patients were excluded because of poor image quality, 6 patients because of previous myocardial infarction and 4 patients because of myocarditis. Among the remaining 24 patients, 14 had mild to severe MR (MR-no MAD) and 10 had no or only a trace MR (no MR-no MAD).

### Baseline clinical and morphological characteristics

Mean age was 50 ± 19 years and similar across the three groups (p = 0.82), and 43% were females. LV end-diastolic volume index was higher in the MVP–MAD group than in the MR-No MAD and the No MR–No MAD groups (102 ± 29 ml/m^2^ vs 81 ± 17 ml/m^2^, p = 0.018, and vs 82 ± 16 ml/m^2^, p = 0.048 respectively), but there was similar LVEF (Table 1). In the MR-No MAD group, MR severity grade assessed by echocardiography was mild in 7 patients (50%), moderate in 5 (36%) and severe in 2 (14%).

**Table 1:** Baseline clinical and cardiovascular magnetic resonance (CMR) characteristics

	MVP–MAD n = 30	Control group (No MVP)			
		MR-NoMAD n = 14	*P vs MVP–MAD*	NoMR-NoMAD n = 10	*P vs MVP–MAD*
Age (y)	50 ± 17	53 ± 22	0.70	46 ± 21	0.53
Male, n (%)	18 (60%)	7 (50%)	0.75	6 (60%)	1.0
Weight (kg)	72 ± 12	70 ± 14	0.69	82 ± 13	0.02
Height (cm)	177 ± 10	170 ± 10	0.04	179 ± 11	0.65
Hypertension, n (%)	2 (7%)	6 (43%)	0.008	2 (20%)	0.26
Active smoker, n (%)	3 (10%)	1 (7%)	1.0	1 (10%)	1.0
Hyperlipidaemia, n (%)	2 (7%)	2 (14%)	0.58	2 (20%)	0.26
LVEDV (ml)	192 ± 56	148 ± 39	0.01	164 ± 29	0.14
LVEDVI (ml/m^2^)	102 ± 29	81 ± 17	0.018	82 ± 16	0.048
LVESV (ml)	84 ± 32	60 ± 21	0.02	70 ± 12	0.20
LVEF (%)	57 ± 8	60 ± 8	0.32	57 ± 3	0.99
LV mass index (g/m^2^)	66 ± 16	62 ± 15	0.45	63 ± 9	0.59
MAD length (mm)	9.4 [7.1–12.3]	–		–	
Presence of LGE, n (%)	14 (47%)	0 (0%)	0.002	0 (0%)	0.007
N of positive segments (n = 14)	3 [3–4]	0		0	
Basal slice pre-Gd T1 (ms)	1067 ± 45	1029 ± 37	0.009	1029 ± 26	0.016
Basal slice ECVsyn (%)	30 ± 3	24 ± 3	< 0.0001	24 ± 2	< 0.0001

*ECVsyn* synthetic extracellular volume fraction, *LV* left ventricle, *LVEDV* left ventricle end-diastolic volume, *LVEDVI* left ventricle end-diastolic volume index, *LVEF* left ventricle ejection fraction, *LGE* late gadolinium enhancement *Gd* gadolinium, *MAD* mitral annular disjunction, *MVP* mitral valve prolapse

In MVP–MAD the maximum MAD length was 9.4 mm [7.1–12.3]. Phase contrast aortic flow data were available in only 22 MVP–MAD patients and the mean mitral regurgitant fraction was 31 ± 17%. After correction for the prolapsing volume, the mean MR fraction was 16 ± 21%. MR severity grade assessed by echocardiography was available in 27 MVP–MAD patients and MR was graded as mild in 9 (50%), moderate in 14 (36%) and severe in 4 (14%) patients.

### Tissue characterization

**Fig. 2:**
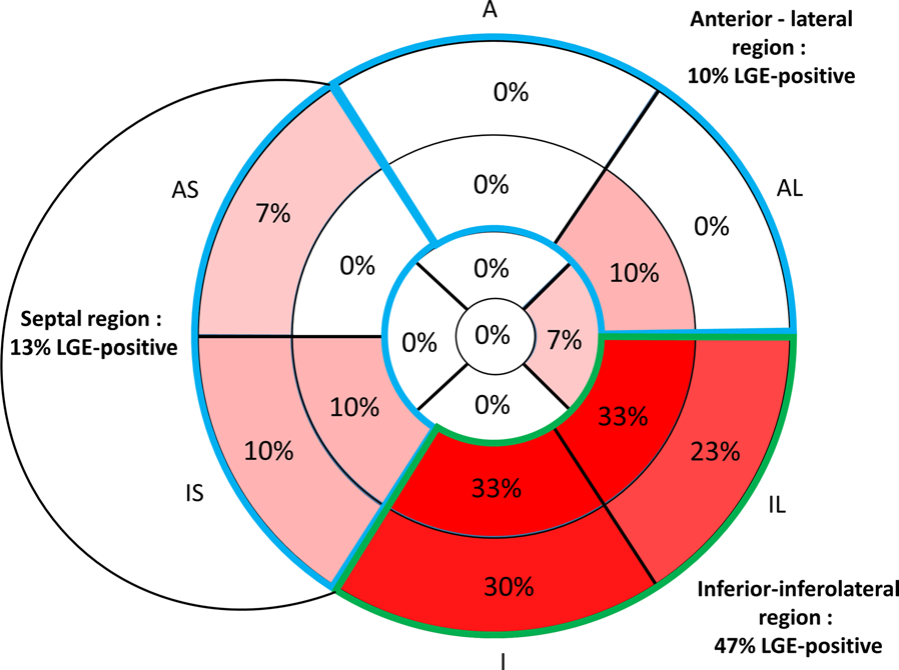
Distribution of late gadolinium enhancement (LGE) positivity in patients with mitral valve prolapse (MVP) and MAD (n = 30). *A* anterior, *AL* anterolateral, *IL* inferolateral, *I* inferior, *IS* inferoseptal, *AS* anteroseptal

LGE was present in 14 MVP–MAD patients (47%) and was absent in the two other groups. In LGE-positive patients, the median LGE extent was 3 [3–4] segments, and LGE was most frequently detected in the basal to mid-ventricular inferior and inferolateral walls (i.e. in the vicinity of the posterior mitral leaflet attachments) compared with the anterior and antero-lateral walls (47% vs 10%, p = 0.002) and the antero-septal and infero-septal walls (47% vs 13%, p = 0.005) (Fig. 2).

**Fig. 3:**
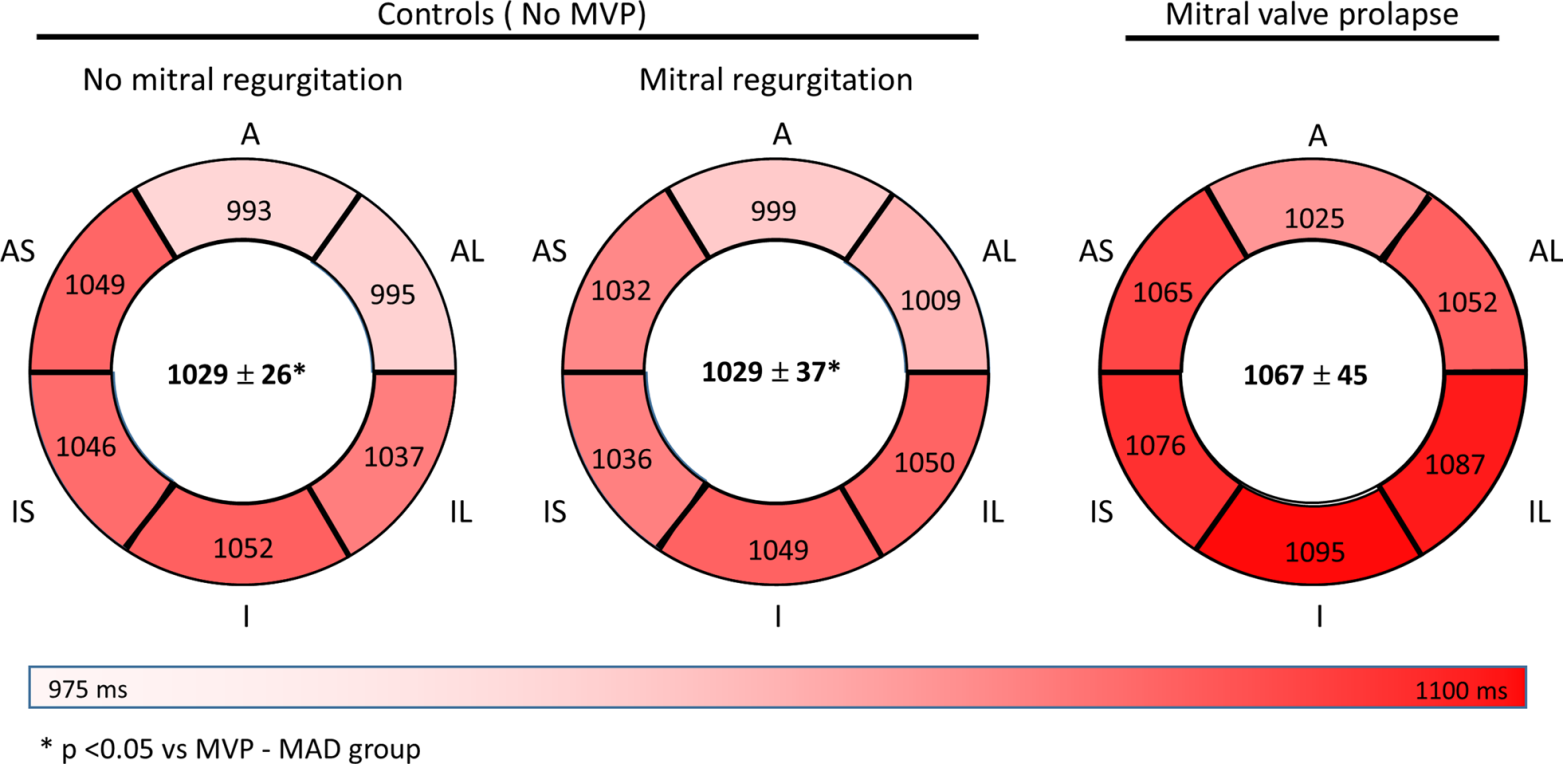
Pre-contrast T1 relaxation time measured in the six basal myocardial segments. The average T1 relaxation time is indicated in the center of each plot. Abbreviations: see Fig. 2

Native T1 relaxation was higher in MVP–MAD in comparison with MR-No MAD (1067 ± 45 ms vs 1029 ± 37 ms, p = 0.009) and No MR-No MAD (1029 ± 26 ms, p = 0.016) (Fig. 3). LGE negative MVP–MAD patients had a native T1 similar to LGE positive MVP–MAD patients (1057 ± 49 ms vs 1078 ± 39 ms, p = 0.19), and significantly higher than controls (1057 ± 49 ms vs 1029 ± 32 ms, p = 0.04).

**Fig. 4:**
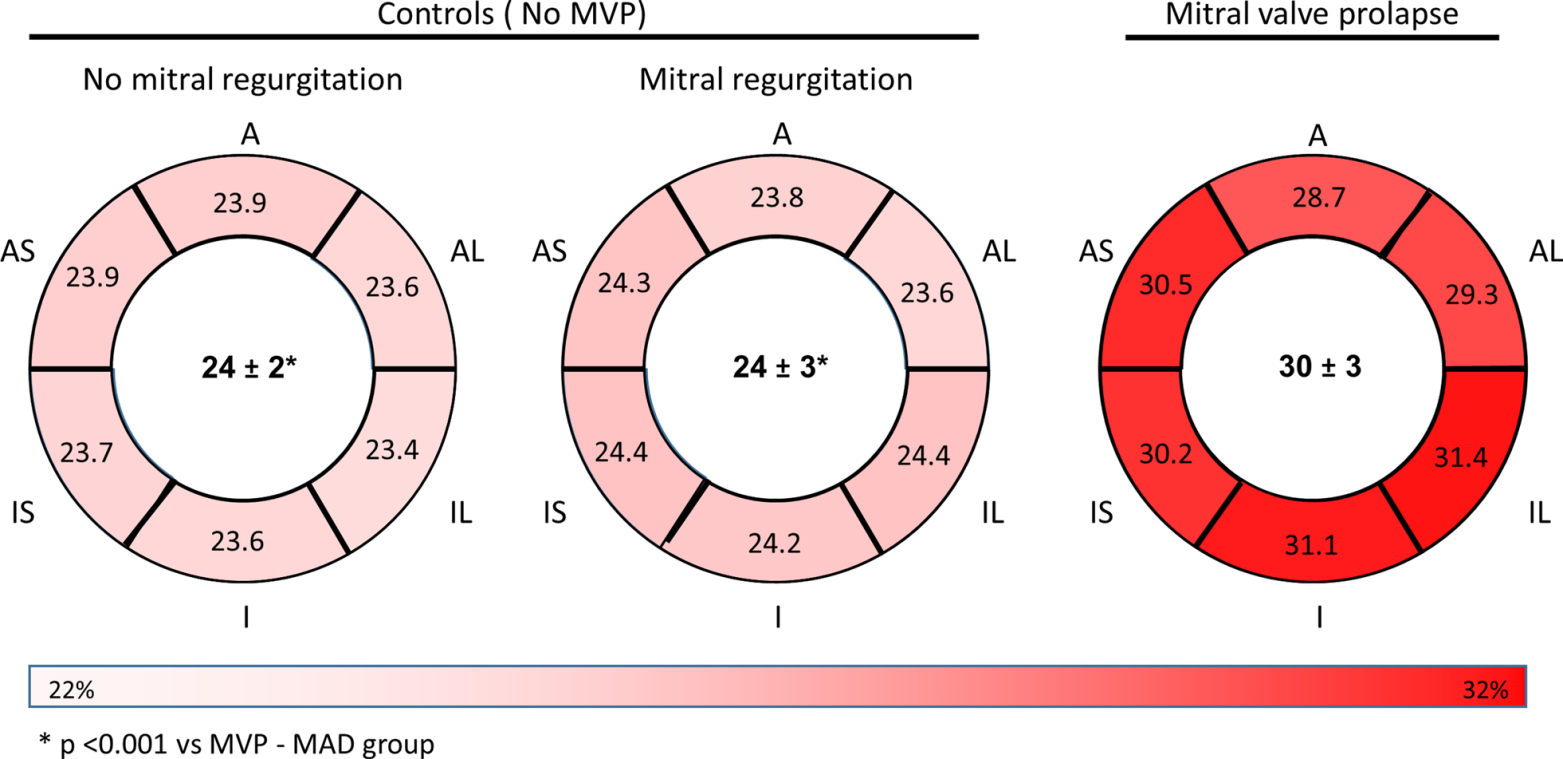
Extracellular volume (ECV) calculated in the six basal myocardial segments. The average ECV is indicated in the center of each plot. Abbreviations: see Fig. 2

The ECVsyn was higher in MVP–MAD compared with MR-No MAD (30 ± 3% vs 24 ± 3%, p < 0.001) and No MR-No MAD (30 ± 3% vs 24 ± 2%, p < 0.001) (Fig. 4). In the MVP–MAD group, LGE negative patients had ECVsyn values similar to LGE positive patients (29 ± 3% vs 31 ± 3%, p = 0.19), and significantly higher than controls (29 ± 3% vs 24 ± 2%, p < 0.001). Interestingly, 87% of the MVP–MAD patients had an ECVsyn above the upper normal limit (> 27%). This proportion was 93% in LGE-positive vs 81% in LGE-negative patients (p = 0.35), and no significant correlation was found between LGE extent and ECVsyn (rho = 0.10, p = 0.60).

**Fig. 5:**
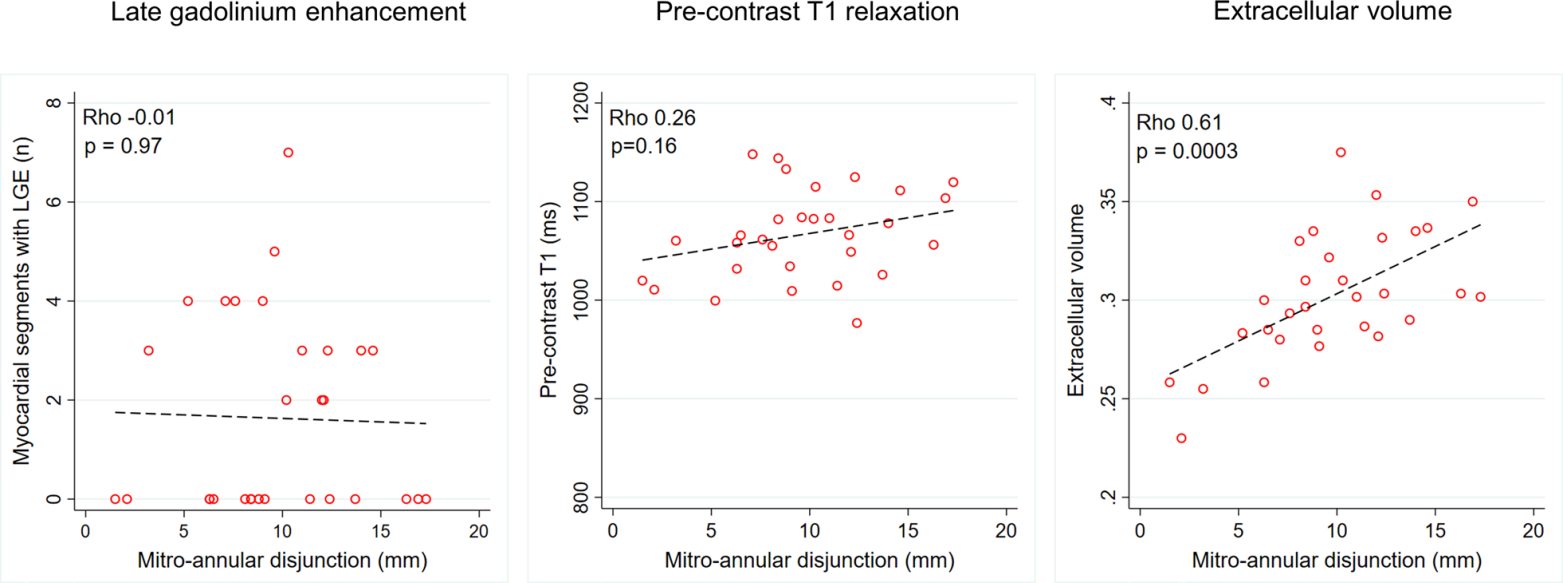
Relation between MAD distance and parameters of myocardial fibrosis

No significant correlation was found between MAD length and LGE extent (rho = − 0.01, p = 0.97) or between MAD length and pre-contrast native T1 (rho = 0.26, p = 0.16) but a moderate to high correlation was found between MAD length and ECVsyn (r = 0.61, p < 0.001) (Fig. 5).

Among the 27 MVP–MAD patients with echocardiographic quantification of MR severity, no significant difference was observed among the three MR severity grades for MAD length (8.4 [7.1–10.2] with mild MR vs 10.9 [9–14.6] with moderate MR vs 8.1 [4.2–12.4] mm with severe MR, respectively, p = 0.16), LGE extent (0 [0–2] vs 0 [0–4] vs 3 [1.5–3.5] segments, p = 0.58), T1 relaxation time (1075 ± 45 vs 1059 ± 44 vs 1042 ± 37 ms, p = 0.50) and ECVsyn (31 ± 5 vs 30 ± 2 vs 28 ± 2%, p = 0.31).

### Associations between, MAD length, myocardial fibrosis, and OHCA

**Fig. 6:**
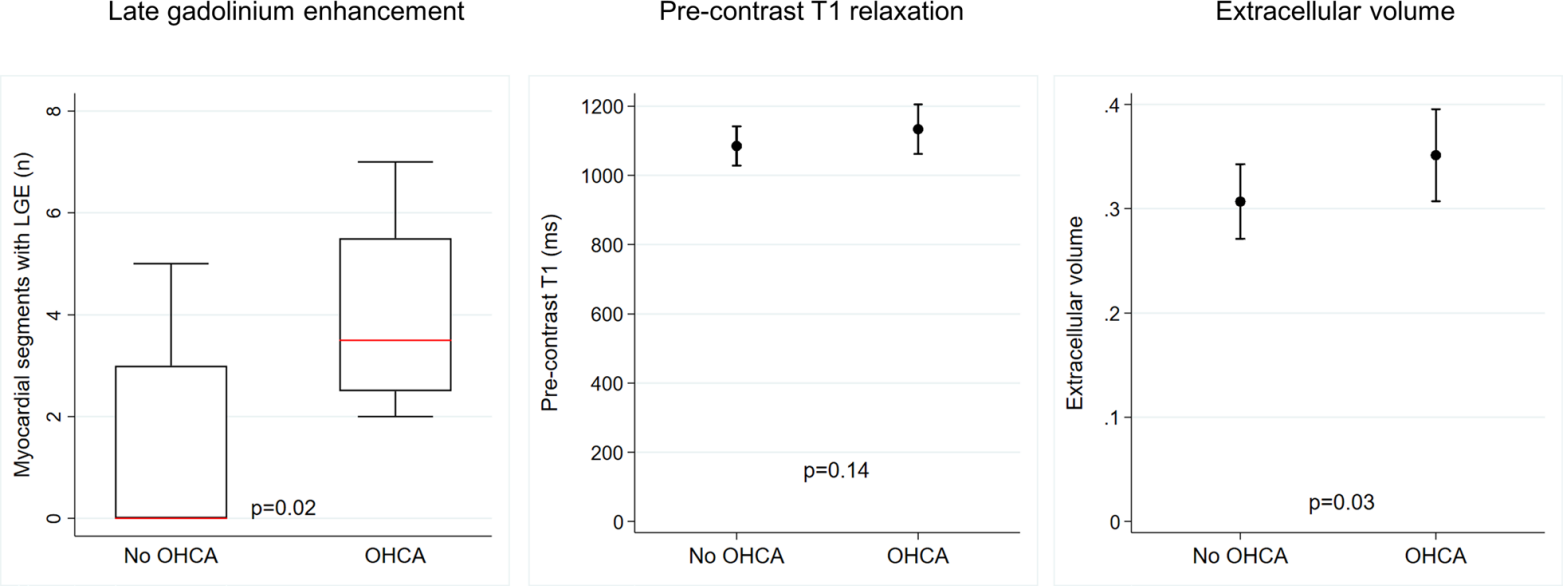
Comparison of fibrosis parameters in patients with and without out-of-hospital cardiac arrest

Among the 30 MVP–MAD patients, 4 (13%) were referred for evaluation following OHCA. None of the patients had evidence of coronary heart disease on coronary angiogram. The clinical characteristics of the 4 patients with history of OHCA are presented in Table 2. Although there was no difference in MAD length (11.2 [8.7–13.0] vs 9.1 [6.5–12.3] mm, p = 0.50), significant differences were found in terms of body weight (85 ± 9 kg in OHCA patients vs 70 ± 11 kg, p = 0.01) and presence of LGE (100% in OHCA patients vs 38%, p = 0.04). OHCA patients had a higher LGE extent (3.5 [2.5–5.5] vs 0 [0–3] segments positive for LGE, p = 0.02). No difference was found in terms of pre-contrast T1 values (1133 ± 72 vs 1085 ± 57 ms, p = 0.14) but a significantly higher ECVsyn was measured in OHCA patients (35.1 ± 4.4% vs 30.7 ± 3.6%, p = 0.03) (Fig. 6). Regarding the identification of patients with history of OHCA, the presence of LGE achieved a sensitivity of 100%, a specificity of 62%, a positive predictive value of 29%, and a negative predictive value of 100%. For an ECVsyn threshold > 33.5%, they were 75%, 85%, 43%, and 96%, respectively. The ROC analysis revealed comparable accuracy of ECVsyn and LGE for the detection of patients with previous OHCA (area under the ROC curve 0.832 vs 0.808, p = 0.84).

**Table 2:** Characteristics of the MVP–MAD patients with and without history of OHCA

	OHCA (n = 4)	No OHCA (n = 26)	p
Age (y)	58 ± 7	49 ± 18	0.33
Male, n (%)	1 (25%)	17 (65%)	0.27
Weight (kg)	85 ± 9	70 ± 11	**0.01**
Height (cm)	178 ± 16	177 ± 10	0.87
LVEDV (ml)	154 ± 16	198 ± 58	0.15
LVEDVI (ml/m^2^)	77 ± 15	106 ± 29	0.06
LVESV (ml)	62 ± 27	87 ± 32	0.15
LVEF (%)	60 ± 14	56 ± 8	0.40
LV mass index (g/m^2^)	60 ± 6	67 ± 17	0.43
MAD length (mm)	11.2 [8.7–13.0]	9.1 [6.5–12.3]	0.50
Presence of LGE, n (%)	4 (100%)	10 (38%)	**0.04**
N of positive segments	3.5 [2.5–5.5]	0 [0–3]	**0.02**
Basal slice pre-Gd T1 (ms)^a^	1133 ± 72	1085 ± 57	0.14
Basal slice ECVsyn (%)^a^	35 ± 4	31 ± 4	**0.03**

Statistically significant correlations are given in bold

*ECVsyn* synthetic extracellular volume, *LV* left ventricle, *LVEDV* left ventricle end-diastolic volume, *LVEDVI* left ventricle end-diastolic volume index, *LVEF* left ventricle ejection fraction, LVESV, left ventricular end-systolic volume. *LGE* late gadolinium enhancement, *Gd* gadolinium, *MAD* mitral annular disjunction, *OHCA* out-of-hospital cardiac arrest

^a^Mean value of inferior and inferolateral segments

Of note, MR was mild in 3 and moderate in one OHCA patient. The mean MR severity grade in OHCA tended to be lower than in patients with no previous OHCA (1.25 ± 0.5 1.91 ± 0.67, p = 0.07), but the difference was not significant.

### Association between myocardial fibrosis and arrhythmia

The baseline characteristics of the 15 patients investigated with a Holter monitoring are presented in Table 3. The median time between Holter monitoring and CMR was 36 days [1–113 days]. The only significant difference between patients with rhythm assessment compared to patients without was a higher ECVsyn of the basal infero-posterior region (33 ± 5 vs 30 ± 3%, p = 0.03). All patients with Holter experienced PVC with a median incidence of 2.8% [1.1–10.3%] of all heartbeats. Complex PVC were detected in 13 patients (87%) and NSVT (non-sustained ventricular tachycardia) in 10 (67%) with a median of 5 [0–17] runs/24 h. LGE was detected in only 8/15 (53%) patients while all patients had an ECVsyn > 27% in the basal infero-posterior region. ECVsyn was not significantly different between patients with and without LGE detected (34.5 ± 5 vs 32 ± 4%, p = 0.47). There was no significant difference in PVC count between patients with and without LGE (4.2% [1.6–8.7%] vs 2.8% [0–14.9%], p = 0.82). Similarly, PVC count was not significantly higher in patients with a higher pre-contrast T1 (T1 ≥ 1115 ms corresponding to the median T1 value of the basal infero-posterior region: 7.1% [1.3–14.9%] vs 2.2% [0.8–7.1%], p = 0.22), and in patients with a higher ECVsyn (ECVsyn ≥ 32.0% corresponding to the median ECVsyn value of the basal infero-posterior region: 2.8% [1.1–10.3%] vs 4.2% [1.2–11.7%], p = 0.77).

**Table 3:** Characteristics of patients with and without Holter monitoring

	Holter (n = 15)	No Holter (n = 15)	p
Age (y)	47 ± 14	53 ± 19	0.34
Male, n (%)	8 (53%)	10 (67%)	0.71
Weight (kg)	74 ± 11	70 ± 13	0.41
Height (cm)	176 ± 7	178 ± 13	0.45
LVEDV (ml)	191 ± 45	193 ± 67	0.93
LVEDVI (ml/m^2^)	102 ± 25	103 ± 34	0.92
LVESV (ml)	85 ± 23	83 ± 39	0.92
LVEF (%)	55 ± 8	58 ± 9	0.33
LV mass index (g/m^2^)	65 ± 10	66 ± 20	0.83
MAD length (mm)	9.6 [7.6–14.6]	9.1 [6.3–11.4]	0.30
Presence of LGE, n (%)	8 (53%)	6 (40%)	0.72
N of positive segments	3 [2.5–4]	3.5 [3–4]	0.59
Basal slice pre-Gd T1 (ms)^a^	1109 ± 49	1073 ± 66	0.11
Basal slice ECVsyn (%)^a^	33 ± 5	30 ± 3	**0.03**

Statistically significant correlation is given in bold

*ECVsyn* synthetic extracellular volume, *LV* left ventricle, *LVEDV* left ventricle end-diastolic volume, *LVEDVI* left ventricle end-diastolic volume index, *LVEF* left ventricle ejection fraction; *LGE* late gadolinium enhancement, *Gd* gadolinium, *MAD* mitral annular disjunction

^a^Mean value of inferior and inferolateral segments

### Intra- and inter-observer reproducibility of T1- and ECVsyn measurements

**Fig. 7:**
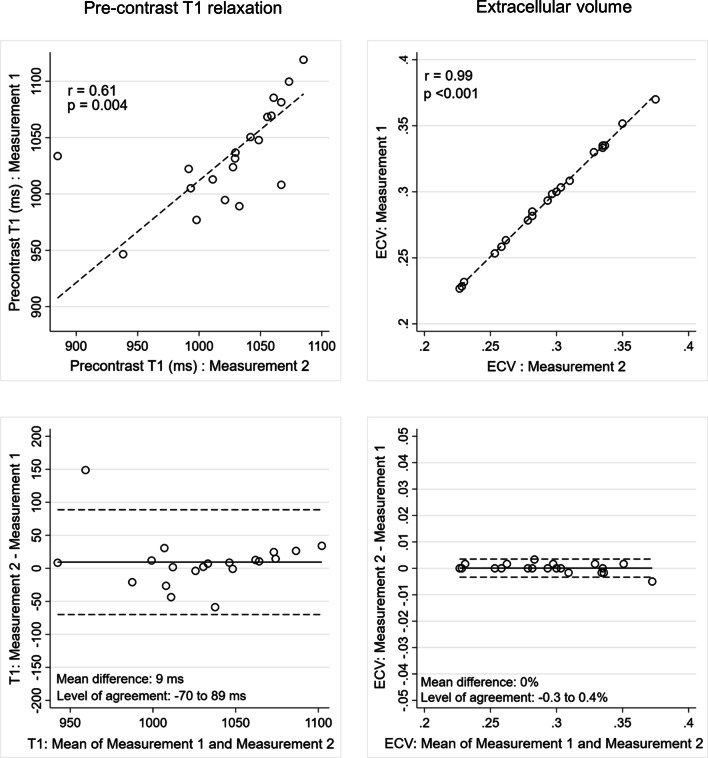
Intra-observer reproducibility of pre-contrast T1 relaxation time (left hand panels) and extracellular volume (right hand panels) measurements

**Fig. 8:**
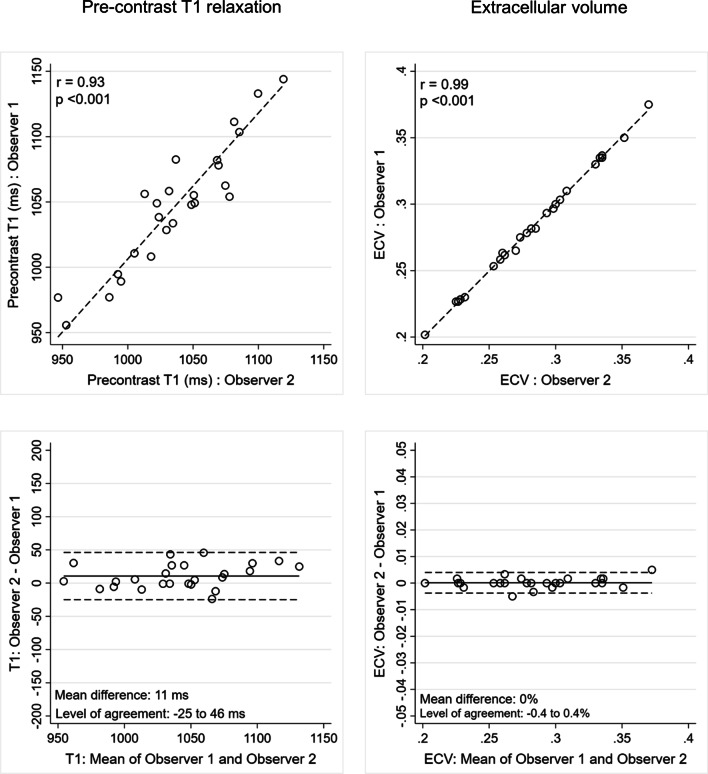
Inter-observer reproducibility of pre-contrast T1 relaxation time (left hand panels) and extracellular volume (right hand panels) measurements

Intra-observer (r = 0.61, p = 0.004/Mean difference 9 ms − 95% levels of agreement—70 to 89 ms) and inter-observer (r = 0.93, p < 0.001/Mean difference 11 ms − 95% levels of agreement—25 to 46 ms) reproducibility for pre-contrast T1 relaxation time was good. Both intra-observer (r = 0.99, p < 0.001/Mean difference 0–95% levels of agreement − 0.3% to 0.4% ms) and inter-observer (r = 0.99, p < 0.001/Mean difference 0% ms − 95% levels of agreement − 0.4% to 0.4%) reproducibility were excellent for ECVsyn calculation (Figs. 7 and 8).

## Discussion

In this retrospective study we found that (1) MVP–MAD patients had a higher ECVsyn indicative of a higher amount of interstitial fibrosis even in the absence of detectable LGE, (2) MAD length was best associated with a higher ECVsyn but not with a higher native T1 or LGE extent, and (3) the presence of LGE and a basal posterior ECVsyn > 33.5% identified MVP–MAD patients with history of OHCA with similar accuracy.

MVP is a relatively frequent clinical condition with a good overall prognosis. However, VA and SCD may occur with an incidence of 0.2% to 0.4% per year in this population [1, 3, 9, 26]. Therefore, the identification of risk factors of VA is of paramount importance. Myocardial fibrosis detected by LGE has been associated with the occurrence of VA in several ischemic and non-ischemic cardiac conditions using CMR [27–30]. However, the precise detection of fibrosis may be challenging in areas where the myocardium is not completely replaced by fibrotic tissue [31] as these inhomogeneous areas are characterized by an intermediate intensity (grey zone) due to the intermingling of both viable myocardium and interstitial fibrosis. They are typically present in the border zone of ischemic scars, or associated with non-ischemic cardiomyopathies or inflammatory heart diseases and may promote re-entry pathways for VA [32–34]. The newer T1 mapping technique provides complementary information on fibrotic remodelling as it allows a quantitative assessment of myocardial ECV, a marker of extracellular matrix expansion [14, 35, 36]. Although increased ECV identifies patients at higher risk of mortality or heart failure [37], its value in predicting adverse arrhythmic outcomes remains unknown.

In patients with MVP and MAD, increasing evidence supports an association between prolapse severity, curling motion of the basal posterior myocardium and structural changes of the myocardium. The continuous mechanical stretch applied by the MVP has been suggested to induce a fibrotic remodelling of the basal inferior LV wall and the papillary muscles [10, 38], where LGE could be detected in 73% and 83% of cases, respectively [10]. MAD itself appears to play an important role in the pathogenesis of VA. In a large cohort of 116 patients with MAD, in which 12% of the population presented with severe arrhythmic events, Dejgaard et al. observed that MAD was a distinct entity linked to arrhythmia severity, whereas isolated MVP was not [11]. Several clinical and pathological studies have revealed an association between bileaflet MVP with MAD, focal fibrosis within the infero-lateral LV segment or papillary muscles, and an increased risk of arrhythmia [6, 7, 10, 21, 26]. Interestingly, in our cohort only 47% of MVP–MAD patients had LGE detected in the LV but 87% had a mean ECV above the upper limit of 27% in the basal LV myocardium. An ECVsyn > 27% was not only found in 93% of LGE-positive, but also in 81% of LGE-negative MVP-MD patients. Moreover, an ECVsyn was increased in all the basal segments, suggesting that fibrotic remodelling is not restricted to the basal inferior/infero-lateral region, but develops in all myocardium adjacent to the insertion of the prolapsing valve. Our findings indicate that detection of confluent fibrosis using LGE alone may underestimate the fibrotic remodelling of the basal myocardium in relation to severe MVP and MAD.

The association between MVP and interstitial fibrosis, and its relation to VA has seldom been investigated. In a series published by Pradella et al. patients with MVP had higher native and lower post-contrast T1 myocardial values, but no association with complex VA was found [17]. Bui et al. also detected an association between MVP and reduced post-contrast T1 times, suggesting increased myocardial fibrosis. Interestingly, in their series, MVP patients with complex VA had lower post-contrast T1 times compared to MVP patients with no arrhythmia making the authors conclude that interstitial remodelling may contribute to ventricular arrhythmogenesis [18]. However both studies did not evaluate the presence of MAD nor calculated the ECV. In the present study, the magnitude of the MAD was strongly correlated to the extent of interstitial fibrosis assessed by ECV, but was not associated with macroscopic fibrosis evaluated by LGE. This suggests that ECV may provide a more comprehensive assessment of LV remodelling induced by MVP–MAD. Based on studies focusing on ischemic heart disease [32–34, 39], we hypothesized that the presence of interstitial fibrosis (i.e. high ECVsyn) could be predictive of VA in MVP–MAD patients. Rhythm assessment was obtained in 15 higher risk patients referred for suspected MVP arrhythmic syndrome and complex VA (either complex PVC or NSVT) were detected in almost all of them. Surprisingly, LGE was detected in only half of the patients but an increase in ECVsyn above the cut-off value was measured in all. Furthermore, ECVsyn was comparably increased in patients with and without LGE, supporting the hypothesis of an implication of interstitial fibrosis, and not only replacement fibrosis, in the development of VA in MVP–MAD patients. Several patterns of remodeling may be linked to the arrhythmic risk in MVP: either focal replacement fibrosis assessed by LGE or interstitial fibrosis measured with T1 mapping. This is in line with the description that VA in MVP can arise from the fascicular region or the septal regions of the mitral annulus in electrophysiological studies, and not only from regions presenting focal macroscopic fibrosis [4]. Finally and most importantly, we found a similar predictive value for OHCA between ECVsyn and LGE extent in our series, which supports systematically performing ECVsyn measurements in addition to LGE assessment during CMR examinations of patients with MVP. This parameter may provide additional prognostic value beyond LGE, as its fair specificity may complement the assessment of LGE in the unmet challenge of primary prevention risk stratification.

Given the retrospective nature of the study, synthetic haematocrit was used to calculate the ECV, as previously described in literature [24, 40, 41]. Until now, some concerns have been raised regarding the reliability of synthetic haematocrit estimations, for instance in the presence of haematologic disorders [42] or in the paediatric population [43], where significant variations between measured and ECVsyn have been observed. Furthermore, the importance of a local calibration of the synthetic haematocrit, which can vary among different scanners, has been highlighted. In our center ECVsyn calculation has been calibrated from a large cohort of patients, no haematologic disorders were present and all patients were > 18 years old. Therefore, the potential inaccuracies associated with this simplified method have been minimized and we believe that the ECVsyn used in this study reflects the true myocardial ECV of the population.

## Limitations

The present study has several limitations. First, it is an observational, retrospective study, including a limited number of patients. Although our population was comparable in terms of arrhythmic MVP. Complex arrhythmia were detected in almost all of them, an evidence of a likely selection bias toward higher arrhythmic risk. Fourth, although we used high resolution bright-blood LGE-CMR and images were interpreted by expert operators, routine evaluation of the presence of fibrosis in the papillary muscles area could prove challenging in routine and was not part of our analysis. Fifth, MR severity was not systematically evaluated during CMR examinations and we cannot assess the influence of MR severity on risk prediction. Sixth, no histologic confirmation of interstitial or replacement fibrosis could be obtained and all our results are based on indirect CMR markers of myocardial fibrosis. Finally, patients underwent to CMR examination in two different scanners (Siemens Aera and Siemens Sola); however local calibration of synthetic haematocrit was performed for both scanners, aim at to obtaining a reliable estimation of the synthetic haematocrit and ECVsyn.

## Conclusion

In patients with MVP–MAD, remodelling of the LV occurs and both focal replacement and interstitial myocardial fibrosis can be detected by CMR. Compared to LGE extent, ECVsyn of the basal LV segments had a stronger association with MAD severity, and a similar association with OHCA. Our data suggest that ECVsyn should be part of the CMR examination of MVP patients in an effort to better assess fibrous remodelling as it may provide additional value beyond the assessment of LGE in the arrhythmic risk stratification.

## Supplementary Information

**Additional file 1: Figure S1. ** Relationship between haematocrit (%) and R1 blood pool (msec^−1^) in the two scanners used in the study (MAGNETOM Aera or Sola, Siemens Healthineers, Erlangen-Germany).

## Data Availability

The datasets used and analysed during the current study are available from the corresponding author on reasonable request.

## Abbreviations

bSSFP: Balanced steady state free precession
CMR: Cardiovascular magnetic resonance
ECG: Electrocardiogram
ECVsyn: Synthetic extracellular volume
LA: Left atrium/left atrial
LGE: Late gadolinium enhancement
LV: Left ventricle/left ventricular
LVEDV: Left ventricular end-diastolic volume
LVEF: Left ventricular ejection fraction
LVESV: Left ventricular end-systolic volume
MAD: Mitral annular disjunction
MOLLI: Modified Look-Locker inversion recovery
MR: Mitral regurgitation
MVP: Mitral valve prolapse
NoMAD: No mitral annular disjunction
NSVT: Non-sustained ventricular tachycardia
OHCA: Out-of-hospital cardiac arrest
PVC: Premature ventricular contraction
SCD: Sudden cardiac death
VA: Ventricular arrhythmia
