# Supplementary figures and images for: Myocardial extracellular volume by T1 mapping: a new marker of arrhythmia in mitral valve prolapse
Source: J Cardiovasc Magn Reson. 2021 Sep 13;23:102. doi: 10.1186/s12968-021-00797-2 (PMC8438990; doi:10.1186/s12968-021-00797-2)

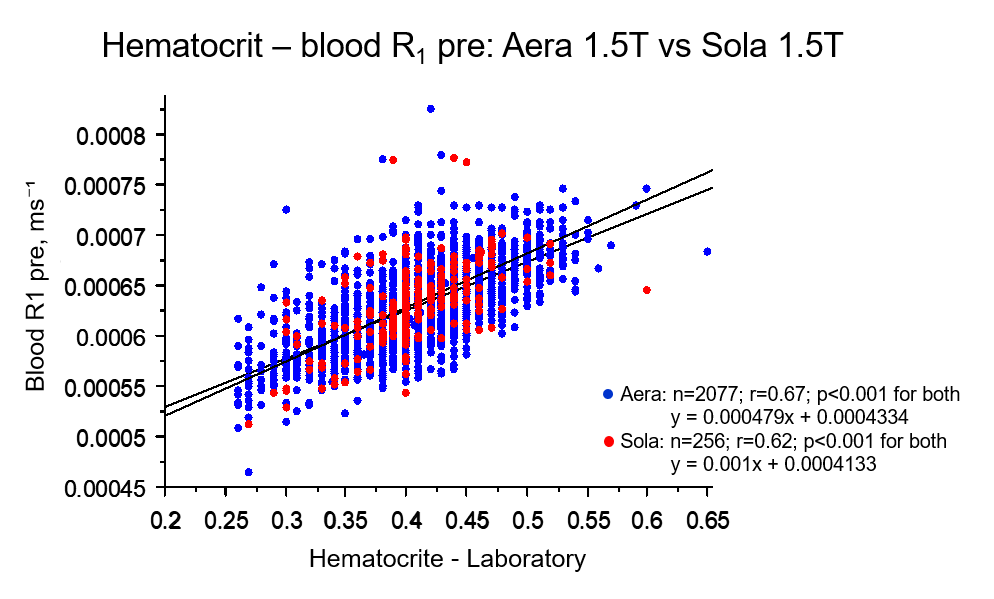

Supplement: Supplementary file 1 — Additional file 1: Figure S1. Relationship between haematocrit (%) and R1 blood pool (msec−1) in the two scanners used in the study (MAGNETOM Aera or Sola, Siemens Healthineers, Erlangen-Germany). [file 12968_2021_797_MOESM1_ESM.tiff]
